# Mitigation of Cadmium Uptake in Bread Wheat (*Triticum aestivum* L.) and Durum Wheat (*Triticum durum* L.) with Natural and Enriched Bentonite Treatments

**DOI:** 10.1021/acsomega.5c00353

**Published:** 2025-03-21

**Authors:** Faruk Özkutlu, Özlem Ete Aydemir, Ayhan Kocaman, Dilek Ece, Mehmet Akgün

**Affiliations:** † Department of Soil Science and Plant Nutrition, Faculty of Agriculture, Ordu University, 52200 Ordu, Turkey; ‡ Engineering Faculty, Environmental Engineering Department, Karabük University, 78050 Karabük, Turkey; § Hazelnut Specialization Coordinatorship, Giresun University, 28100 Giresun, Turkey

## Abstract

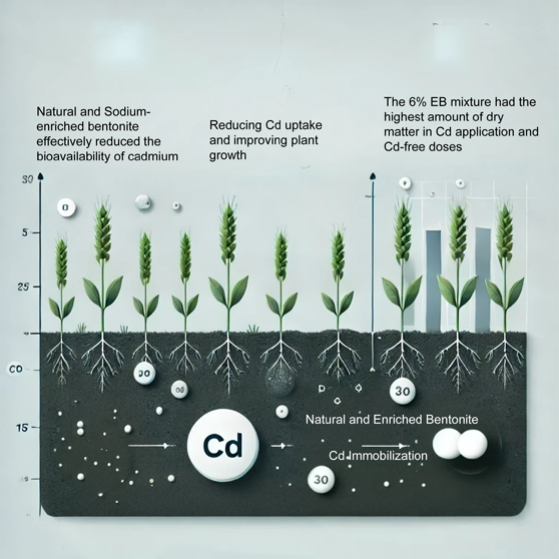

Soil pollution by heavy metals is a significant issue impacting food security and human health. Cadmium, a toxic metal, contaminates soils via industrial and agricultural activities, posing risks to the food chain. This study aimed to evaluate methods for reducing cadmium bioavailability in bread wheat and durum wheat, crucial crops for human nutrition grown on contaminated soils. A greenhouse experiment was conducted in which soil samples were treated with 3–6% natural bentonite and sodium-enriched bentonite and contaminated with 5 and 10 ppm cadmium. Compared to controls, cadmium bioavailability in bread wheat decreased by 55% with 5 ppm of Cd and by 66% with 10 ppm of Cd when treated with 6% sodium-enriched bentonite. Similarly, in durum wheat, cadmium bioavailability decreased by 55% and 48% at 5 and 10 mg Cd kg^–1^, respectively. Additionally, 6% natural and enriched bentonite applications increased biomass production in both wheat varieties. Bread wheat dry matter increased by 43.69% with 5 ppm of Cd and natural bentonite, while durum wheat showed an increase of 88.66% with 10 ppm of Cd and enriched bentonite. In bread wheat, the highest B concentration was obtained with 6% NB at 5 and 10 ppm of Cd, with increases of 15.5%, 39.53%, and 16.56% compared to controls; similar increases were seen in durum wheat. Ca concentrations increased with Cd application in control samples, whereas Mn concentrations decreased with Cd and bentonite treatments. The highest Na concentrations in both wheat varieties were recorded at 6% EB, resulting in significant increases (bread wheat: 2434%–4126%; durum wheat: 2763%–3592%) compared to controls. Nutrient stability for Fe, Cu, K, Mg, P, and Zn varied according to Cd dose and bentonite type. The addition of natural and sodium-enriched bentonite effectively reduced cadmium bioavailability in bread and durum wheat, while promoting increased biomass production. These findings suggest that bentonite amendments have potential applications for enhancing crop yields and ensuring food safety in cadmium-contaminated environments.

## Introduction

Heavy metal pollution of the soil is a critical global environmental problem, affecting over 10 million sites and contaminating more than 50% of agricultural land.^1,2^ Key anthropogenic contributors to heavy metal accumulation include mining, smelting, industrial waste discharge, fossil fuel combustion, military activities, and electronic manufacturing.^3−5^ Additionally, excessive reliance on chemical fertilizers in agriculture has significantly exacerbated this contamination.^6^

Soil screening guidelines and risk intervention levels show that heavy metal levels significantly exceed the standard limits set in the United States. Similarly, environmental quality assessments in China show alarmingly high levels of pollution.^2,7^ Studies also show that soils in European and American cities are moderately to highly contaminated, with elevated concentrations of Cd, Zn, Cu and Pb compared to cities in Africa and Asia.^8−10^ In contrast to organic pollutants, heavy metals remain in the soil indefinitely as they are neither microbially nor chemically degraded. Consequently, their concentrations and toxicity remain unchanged long after their initial introduction.^11,12^ The risks that these pollutants pose to human health and the environment depend not only on their overall concentration, but also on their mobility within the soil system.^13,14^

Heavy metal pollution in agricultural areas is of particular concern, as the transfer of these metals from the soil into the food chain significantly increases the risk to human health.^15−20^ The toxicity of metals is a widely recognized environmental health problem that poses significant risks due to its potential for bioaccumulation in the food chain, which can lead to dangerous consequences in humans and animals.^21,22^ In many developing countries, a large part of the population is confronted with problems related to food contamination with toxic metals. In many developing countries, a large proportion of the population is confronted with the problem of food contamination by toxic metals. This problem arises from the inadequate regulation of sources of toxic metals and the uptake of metals by plants in high concentrations.^23−25^ Due to their persistence and widespread occurrence, these metals can enter the human body and disrupt cellular processes by impairing essential metal functions. Cd, for example, is a toxic metal commonly found in the environment. It has no known beneficial function for humans and has no homeostatic regulatory mechanism.^25^

Oxidative damage to biological macromolecules is often associated with the binding of metals to DNA and nuclear proteins.^26^ The severity of the symptoms of metal poisoning varies depending on the level of exposure. In humans, it can lead to mental retardation in children, dementia in adults, central nervous system disorders, kidney and liver disease, insomnia, emotional instability, depression and visual disturbances.^27^ At higher exposure levels, Cd toxicity primarily affects vital organs, including the liver, kidneys, placenta, brain, lungs and bones, and has been associated with serious illnesses such as pulmonary edema, emphysema, bronchiolitis, alveolitis and even death.^28,29^

The extent of cadmium exposure and the associated health risks vary considerably between different population groups. These differences are primarily due to differences in dietary habits and environmental pollution. In Europe, for example, daily Cd intake is between 10 and 18 μg/day, with the main sources being cereals, potatoes, vegetables and seafood. In Asia, countries such as Japan (26.4 μg/day) and Vietnam (109–122 μg/day, high exposure) have an increased Cd intake due to rice consumption. Thailand records extreme values, with men reaching 188–224 μg/day. In the Americas, Cd intake is 10.4 μg/day (USA), 18.12 μg/day (Chile) and 24.8 μg/day (Canada).^30^ These dietary sources contribute significantly to Cd-related health risks, especially in high-exposure regions. Given the high dietary exposure to Cd in certain populations, cadmium toxicity has been closely associated with several clinical conditions, including heart failure, cancer, anosmia, osteoporosis, cerebrovascular infarcts, proteinuria, cataracts, and emphysema.^31^ Cd poses a significant risk to human and animal health as it can accumulate in edible plant parts such as fruits and seeds and thus enter the food chain. In addition, Cd is an ecotoxic element that negatively affects the biological functions of plants.^32^ Its bioaccumulation in plants is influenced by factors such as bioavailability in the soil, the soil’s pH value, and the plant’s genotype.^33^

Certain plant species can tolerate Cd accumulation through mechanisms such as compartmentalization, redox homeostasis maintenance, reduced rhizosphere Cd activity, and limited Cd translocation to aboveground tissues. In contrast, severe morphological, physiological, biochemical, and molecular disturbances occur in Cd-sensitive plants.^34,35^ Studies have shown that Cd toxicity inhibits seed germination, inhibits plant growth, reduces root development and decreases leaf production, ultimately leading to plant death.^36−38^ Cadmium has also been reported to cause physiological disorders in plants, particularly due to oxidative stress triggered by the overproduction of reactive oxygen species (ROS).^39^ As a highly toxic metal, Cd presents significant risks to human, animal, and plant health. It enters the environment mainly through anthropogenic sources, including agricultural chemicals (fertilizers and pesticides), industrial activities, and mining.^40−44^ Phosphate fertilizers contribute to Cd contamination of agricultural soils, as their excessive use increases the amount of bioavailable Cd, accumulating in plants and entering the food chain, posing a risk to human health.^45−48^

Although various remediation techniques exist, conventional methods are often expensive, harmful to the environment, and impractical for large-scale applications. Immobilization, an alternative approach, reduces the bioavailability of Cd through adsorption and precipitation reactions and limits plant uptake.^49^ Additionally, clay minerals, oxides and hydroxides of Al, Fe and Mn,^46^ such as nano-Fe, Zinc oxide, Magnesium oxide,^47^ have been used for metal immobilization due to their high absorption capacity.^48^ Organic materials such as chicken manure and vermicompost are also used.^49^ Bentonite, a type 2:1 aluminosilicate, consists mainly of montmorillonite and is characterized by its high permanent negative charges and large specific surface area. It has proven an effective adsorbent, especially for heavy metals such as Pb and Cd.^50−52^ Bentonite is a nonmetallic mineral whose main component is montmorillonite.^53^ Due to its excellent adsorption and cation exchange properties,^54^ it can also be used in other areas, such as wastewater treatment and soil remediation.^55,56^ Clay minerals such as bentonite and zeolite consist mainly of aluminum oxide and silicon dioxide. Due to their large specific surface area, they are often used as metal sorbents for the remediation of metal-contaminated soils.^57^ Bentonite, for example, has a high sorption capacity for Cd and Pb in the soil, immobilizing these metals and reducing their plant uptake.^58−60^ Since bentonite is naturally negatively charged, it can adsorb positively charged metal ions such as Cd on its surface. These negative charges attract Cd ions, leading to their immobilization on the surface, thus reducing their bioavailable forms in the soil.^61,62^

The bioavailability of Cd from food is a key factor in determining its potential risk.^63^ In soil, the toxicity of metals is primarily influenced by their bioavailability, which refers to the proportion of a pollutant in interstitial water and soil particles that organisms can take up. It determines how much of a nutrient, toxicant, or other substance is available for uptake and accumulation after exposure.^64^ In one study, the addition of bentonite decreased the bioavailability of Cd by increasing adsorption, ion exchange, and complexation reactions in soil, decreasing Cd concentrations in edible parts of vegetables.^65^ Bentonite has also been reported to reduce the uptake of Cd in rice, corn, and vegetables.^55,58,66−72^ Combined inorganic and organic modifications generally have high cation exchange capacity and greater affinity for chelating groups and trace elements. In this way, their transfer from the soil to the roots is prevented, thus reducing their accumulation in the shoots.^73,74^

In this study, bread wheat (*Triticum aestivum* L.) and durum wheat (*Triticum durum* L.) were selected as target crops due to their status as staple foods and their susceptibility to Cd uptake. Their widespread consumption, especially in regions with a high risk of Cd contamination such as Europe, North America and Asia, increases the likelihood of Cd entering the human food chain, making wheat an important topic of this study. This study aimed to reduce the bioavailability of Cd in wheat by using natural and sodium-enriched bentonite to immobilize Cd in contaminated soils.^73^ Bentonites were chosen for their high adsorption capacity, cost-effectiveness, and efficiency in the removal of pollutants.^75^ Due to its higher cation exchange capacity, sodium-enriched bentonite was used to evaluate its effects on the immobilization of Cd in soils. Its application in Cd-contaminated soils aims to reduce Cd mobility, improve nutrient availability, and support plant productivity.

## Material and Method

### Experimental Design

The experiment was conducted in pots under greenhouse conditions at the Faculty of Agriculture of Ordu University (40°58′04″ N, 37°56′17″ E, altitude: 8 m above sea level). The trial was conducted in a randomized factorial design with 3 Cd doses (0, 5, and 10 mg kg^–1^), 2 types of bentonite [natural bentonite (NB) and Sodium-enriched bentonite (EB)], and 3 bentonite application rates (0%, 3% and 6%). In addition, two species (*T. aestivum* L. and *T. durum* L.) were used. Each treatment was replicated three times (with three pots per replicate), resulting in a total of 108 pots. To assess the effects of these treatments, wheat was grown in soils NB and EB with 3% and 6% bentonite and contaminated with 5 and 10 mg Cd kg^–1^. After harvest, Cd and nutrient concentrations in the biomass, as well as Cd immobilization and plant productivity parameters, were analyzed.

### Plant Material and Growth Conditions

The greenhouse conditions were climate-controlled, with a day/night temperature of 22/14 °C, a relative humidity of 70 ± 3%, and an illumination of 55,000 lx with a 14 h day and 10 h night cycle. In this experiment, *Bayraktar* was used as a species of bread wheat (*Triticum avestium* L.) and *Mirzabey* as a species of durum wheat (*T. durum* L.). The wheat varieties were obtained from the Seed Research Institute in Ankara. For the experiment, 1.7 kg of air-dried soil sieved through a 4 mm sieve was placed in each pot. Initially, 12 seeds were planted in each pot, and after emergence, when the seedlings reached the two-leaf stage, they were decimated to eight plants per pot. The pots were irrigated daily with deionized water to keep the soil moisture between 60% and 80% of its water holding capacity.

### Cadmium Treatments and Bentonite Applications

The soils used for the experiment were contaminated with two different amounts of Cd (3CdSO_4_·8H_2_O) at a dosage of 5 and 10 mg kg^–1^. In addition, natural bentonite (NB) and enriched bentonite (EB) were incorporated in quantities of 3% and 6%, respectively. A control group without Cd and bentonite was also used for comparison. The Cd, NB, and EB doses were thoroughly mixed into the soil to ensure homogeneity.

### Fertilization

The basic fertilizer applied was 200 mg N kg^–1^ (from Ca(NO_3_)_2_·4H_2_O) and 100 mg P kg^–1^ (from KH_2_PO_4_) per kg of soil. Fertilization was carried out in a single application, with the nutrients distributed evenly across all pots.

### Plant Harvesting, Sample Preparation, and Elemental Analysis

After 45 days of growth in the greenhouse, the plants were harvested. The harvested plants were dried at 65 °C for 48 h, and the dry matter yield (mg plant^–1^) was determined. The dried plant samples were crushed using the agate mill after weighing the results. Of the samples of shoot, 0.25 g were weighed. Then, 2 mL of distilled water, 2 mL of H_2_O_2_ (30%) and 4 mL of HNO_3_ (65%) were mixed and burned in microwave (CEM MARS 6). After combustion, the samples were cooled to ambient temperature and then made up to 25 mL with distilled water and filtered with blue band filter paper. Ca, P, K, Mg, B, Cu, Mn, Zn and Cd concentrations in these filters were measured by inductively coupled plasma atomic emission spectrometry (ICP-OES; Varian Vista-pro). To confirm the accuracy of the analyzes, standard reference materials (peach leaves, SRM 1547) certified by the National Institute of Standards and Technology (Gaithersburg, MD, USA) were used and the deviations were determined to be less than 1–3%.

#### Physical and Chemical Parameters of the Soil Used for the Study

The soil used for the experiment was classified as moderately acidic, loamy and slightly calcareous, the organic matter content (OM) is low, there is no salinity, the P concentration is insufficient, and the K concentration is adequate (Table 1). The physical and chemical properties of experimental soils were determined using methods by standard methods given in Jackson.^76^ Total nitrogen (N),^77^ available P,^78^ exchangeable K, Ca, and Mg analyzes according to the 1N Ammonium Acetate (pH = 7) method,^76^ soil pH and electrical conductivity (EC) were analyzed in 1:2.5 soil-water extracts.^79^ Soil organic matter and lime^76^ were tested, available Zn, Fe, Cu and Mn was made according to Lindsay and Norvell.^80^ The National Institute of Standards and Technology (Gaithersburg, MD, USA) certified standard reference materials were used to verify the accuracy of element analyses.

**Table 1:** Physical and Chemical Properties of the Soil and Bentonite Used in the Study

soil		bentonite		
			enriched bentonite	natural bentonite
texture	loamy	SiO_2_ (%)	68.1	65.61
pH (1:2.5)	4.81	Al_2_O_3_ (%)	16.5	14.17
EC (μs/cm)	652	Fe_2_O_3_ (%)	1.1	1.22
calcareous (%)	0.8	TiO_2_ (%)	0.14	0.12
OM (%)	1.52	CaO (%)	1.9	3.11
N (%)	0.14	MgO (%)	3.2	2.07
P (mg kg–1)	3.74	Na_2_O (%)	1.5	0.99
K (mg kg–1)	222	K_2_O (%)	1.1	1.5
Ca (mg kg–1)	1334	the loss of radiate heat (%)	6.5	4.7
Mg (mg kg–1)	166	cation exchange capacity (Meq/100 gr)	96,2	85
Fe (mg kg^–1^)	13.5	distention (2 g)	25 mL	9
Zn (mg kg^–1^)	4.44	pH	10–12	7–9
Mn (mg kg^–1^)	21.26	EC (μs/cm)	370	180
Cu (mg kg^–1^)	2.48	calcif (%)	0.3	0.8
Cd (mg kg^–1^)	0.51			

### Physical and Chemical Properties of the Bentonite Used for the Experiment

The bentonite for this experiment was provided by Bentaş Bentonite Mining Company. The natural and enriched bentonites used (enriched with 3% sodium carbonate) were dried (maximum moisture content 10%) and sieved to a thickness of 2 mm (Table 1). An inorganic natural material, aluminosilicate 2:1 type bentonite,^73^ was used. It has a crystalline structure consisting of an octahedral layer of alumina between two layers of tetrahedral silicon with high permanent negative charges, and it also has a large specific surface area. The expanding layers of aluminosilicates with various exchangeable surface cations, such as calcium, sodium, magnesium and potassium, provide affinity and capacity for the adsorption of bentonite.

#### Statistical Analyses

Data were analyzed using two-way ANOVA in SPSS software (version 22.0). Duncan’s Multiple Range Test was performed to determine significant differences between means (p < 0.05). Data followed by different letters are significantly different according to Duncan’s Multiple Range Test (p < 0.05). Values without letters indicate no significant difference (*p* > 0.05).

## Results

### Bread Wheat

**Figure 1:**
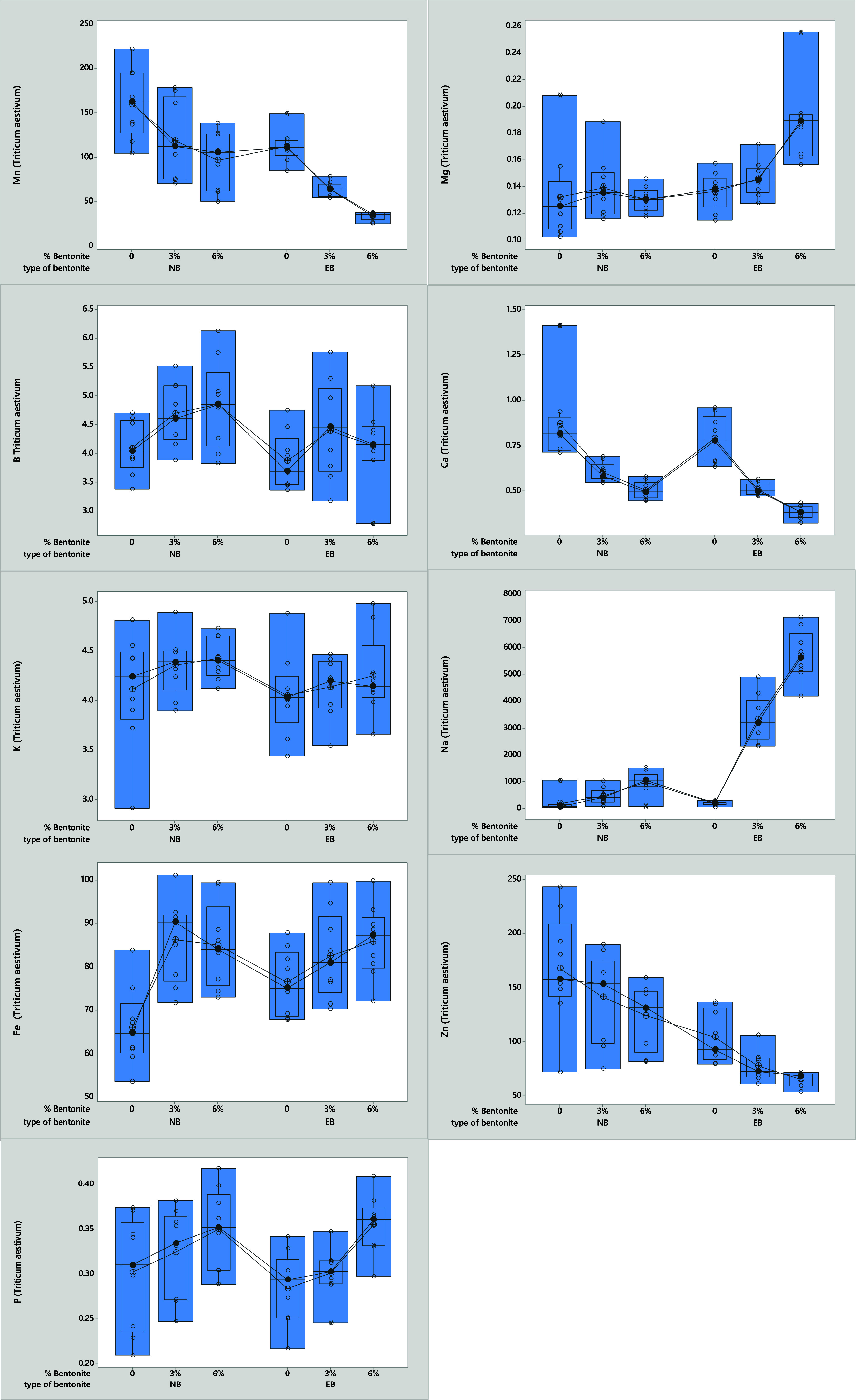
Effect of bentonite type and ratio on Cd accumulation in the shoot of *Triticum aestivum* L..

**Figure 2:**
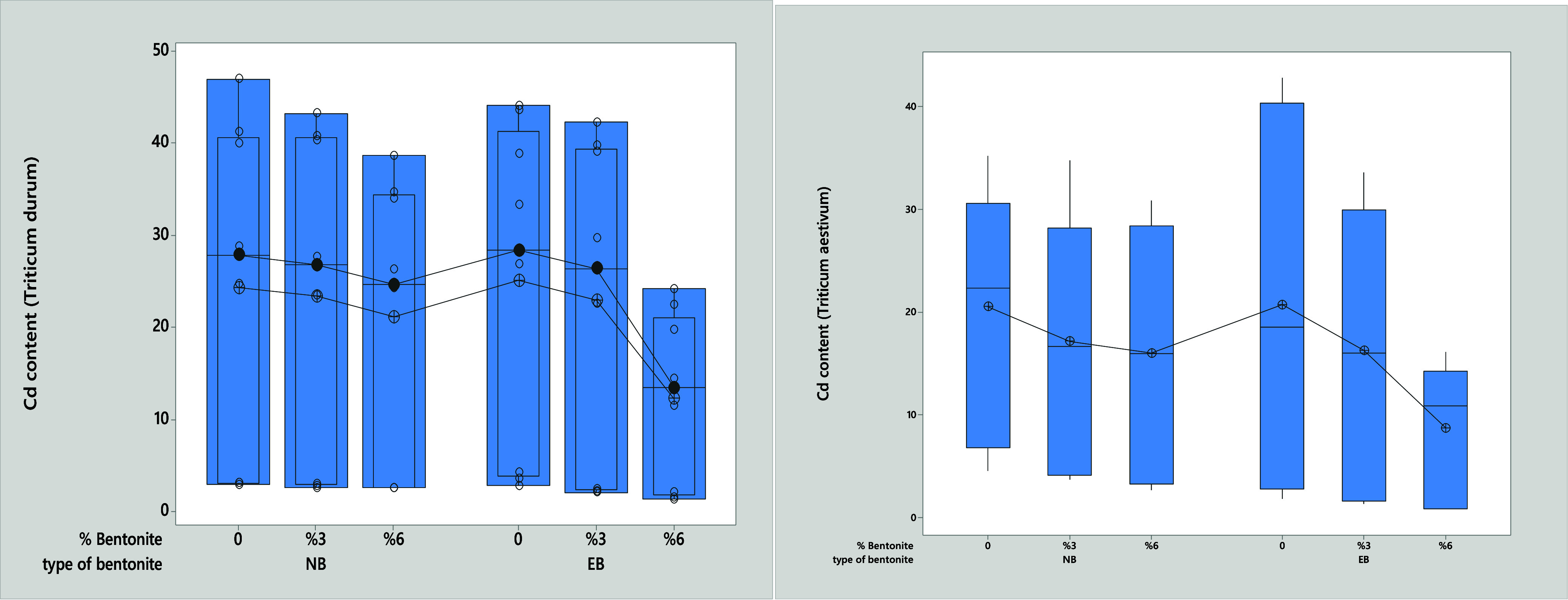
Effect of bentonite type and ratio on Cd concentration in both wheat varieties.

As shown in Table 2, there was a statistically significant difference in the effect of Cd applications on the nutrient concentration of bread wheat (p < 0.05), except for the B and Ca contents in the Cd0 groups. The highest B concentration was obtained at 6% NB (5.33–5.21 mg plant^–1^). The highest Ca % concentration as a function of Cd doses and bentonite mixture ratio were obtained in the control applications and were statistically significant (0.75-0.86-1.00) (p < 0.05). While there were statistically significant differences between the dosage of 5 mg Cd kg^–1^ in different bentonite mixtures and plant Cu concentration, there was no statistically significant difference in the application of 10 mg Cd kg^–1^ (p < 0.05). The highest plant Cu concentration was obtained at 6% EB with a dose of 5 mg Cd kg^–1^ (9.95 mg kg^–1^). At Cd-free doses, the highest Cu concentration was also reached at 6% EB (9.48 mg kg^–1^). In contrast, the highest Cu concentration was obtained at a Cd dose of 10 mg kg^–1^ in the control group (9.32 mg kg^–1^). The highest Fe concentration was obtained at 6% EB in the Cd-free group (93.37 mg kg^–1^), while it was highest in the 3% mixture NB at a Cd dose of 5 ppm (88.84 mg kg^–1^). On the other hand, the highest Fe concentration was obtained in the 3% EB at a Cd dose of 10 mg kg^–1^ (82.57 mg kg^–1^). The highest K concentration in the plant samples was obtained with 6% NB and Cd-free application (4.39%). At a Cd dose of 5 mg kg^–1^, the highest K concentration was obtained with 6% EB (4.67%) and at a dose of 10 mg kg^–1^ with 6% NB (4.48%). The highest Mg concentrations were obtained by the plants in the 6% EB mixture at the applications of 0, 5, and 10 (mg kg^–1^) compared to the other groups. They were respectively (0.18–0.20–0.18 mg kg^–1^). The highest Mn concentration was obtained in the control groups (203.59-–156.26–119.38 mg kg^–1^). The highest Na concentration was found in the 6% EB mixtures (5853.41–5503.66–5790.87 mg kg^–1^). The highest P concentration was observed in the 6% NB mixture at Cd doses of 0 and 5 mg kg^–1^ (0.39–0.37%). In contrast, the highest P concentration was observed in the 6% EB mixture (0.35%) at a Cd dose of 10 mg kg^–1^. Zinc concentrations were significantly higher in the control groups and ranged from 163.88 to 220.28 mg kg^–1^ and 132.89 mg kg^–1^ (Figure 1). As shown in Figure 2, the Cd concentrations in the plants varied significantly depending on the ratio of the bentonite mixture (p < 0.05). The highest Cd accumulation was recorded in the control groups (6.10–23.38–41.16 mg kg^–1^), while the lowest Cd concentrations were observed in the 6% EB mixture (0.86–10.53–14.89 mg kg^–1^).

**Table 2:** Shoot Concentrations of Macro, Micronutrients, and Cadmium^a^

	% bentonite and type	Triticum aestivum L			Triticum durum L		
		0	5	10 mg Cd kg^–1^	0	5	10
B mg kg–1	0	3.98 ± 0.58	3.82 ± 0.17bc	4.47 ± 0.32ab	3.92 ± 0.81	3.35 ± 0.47ab	3.08 ± 0.71b
	% 3NB	4.57 ± 0.85	4.73 ± 0.52ab	4.79 ± 0.34ab	3.85 ± 1.40	3.57 ± 0.39ab	3.57 ± 0.69ab
	% 6NB	4.03 ± 0.22	5.33 ± 0.69a	5.21 ± 0.49a	3.97 ± 0.25	4.14 ± 0.60a	4.19 ± 0.39a
	0	4.13 ± 0.55	3.43 ± 0.06c	4.06 ± 0.40ab	3.39 ± 0.67	2.67 ± 0.69b	2.57 ± 0.44b
	% 3EB	4.77 ± 0.64	4.03 ± 0.75bc	4.38 ± 1.20ab	4.14 ± 1.20	3.00 ± 0.20b	3.00 ± 0.77b
	% 6EB	4.64 ± 0.46	4.19 ± 0.33bc	3.56 ± 0.69b	2.54 ± 0.29	2.86 ± 0.64b	2.67 ± 0.39b
Ca %	0	0.75 ± 0.06	0.86 ± 0.07	1.00 ± 0.37	1.12 ± 0.10a	1.24 ± 0.09a	1.15 ± 0.02a
	% 3NB	0.60 ± 0.07	0.58 ± 0.02	0.63 ± 0.06	1.15 ± 0.29a	1.00 ± 0.07*b	1.03 ± 0.09ab
	% 6NB	0.47 ± 0.02	0.50 ± 0.03	0.53 ± 0.07	0.73 ± 0.08b	0.93 ± 0.06bc	0.96 ± 0.11b
	0	0.73 ± 0.12	0.73 ± 0.08	0.91 ± 0.07	1.23 ± 0.10c	1.20 ± 0.04a	1.05 ± 0.06ab
	% 3EB	0.51 ± 0.042	0.50 ± 0.03	0.52 ± 0.04	0.81 ± 0.04b	0.79 ± 0.13c	0.79 ± 0.02c
	% 6EB	0.39 ± 0.03	0.41 ± 0.03	0.35 ± 0.03	1.48 ± 0.00a	0.53 ± 0.04d	0.51 ± 0.04d
Cd mg kg–1	0	6.10 ± 1.49a	23.38 ± 1.89a	32.14 ± 3.26b	3.00 ± 0.10ab	27.10 ± 2.17a	42.69 ± 3.74a
	% 3NB	4.04 ± 0.24b	17.10 ± 1.21bc	30.37 ± 4.33b	2.78 ± 0.23bc	25.92 ± 2.21a	41.43 ± 1.56a
	% 6NB	3.10 ± 0.35bc	15.74 ± 0.44c	29.23 ± 2.39b	2.53 ± 0.02bc	25.03 ± 1.11a	35.76 ± 2.49b
	0	2.51 ± 0.66 cd	18.56 ± 0.84b	41.16 ± 1.74a	3.48 ± 0.70a	29.46 ± 3.35a	42.15 ± 2.89a
	% 3EB	1.56 ± 0.15de	16.15 ± 1.65c	31.15 ± 2.76b	2.22 ± 0.19c	26.20 ± 3.56a	40.30 ± 1.72a
	% 6EB	0.86 ± 0.04e	10.53 ± 0.81d	14.89 ± 1.59c	1.59 ± 0.37d	13.05 ± 1.51b	22.08 ± 2.24c
Cumg kg–1	0	6.10 ± 0.49d	6.49 ± 0.53c	7.03 ± 0.07b	8.22 ± 0.63bc	9.27 ± 0.65b	9.94 ± 0.49
	% 3NB	6.14 ± 0.36d	6.30 ± 0.57c	7.52 ± 0.62b	8.87 ± 1.83abc	9.61 ± 1.27ab	10.60 ± 0.40
	% 6NB	5.85 ± 0.09d	6.41 ± 0.39c	7.87 ± 0.52c	7.50 ± 0.87c	10.24 ± 0.38ab	10.48 ± 1.35
	0	7.58 ± 0.31*c	8.02 ± 0.32b	9.32 ± 1.08a	7.22 ± 0.71c	11.08 ± 0.00a	11.35 ± 0.79
	% 3EB	8.45 ± 0.04b	7.75 ± 0.93b	7.83 ± 0.34b	9.69 ± 0.49ab	9.90 ± 1.37ab	11.24 ± 0.89
	% 6EB	9.48 ± 0.37a	9.95 ± 0.89a	8.31 ± 0.83ab	10.33 ± 0.50a	9.98 ± 0.33ab	11.03 ± 0.45
Femg kg–1	0	64.40 ± 2.84c	75.64 ± 7.94b	57.97 ± 3.89b	62.29 ± 0.90bc	54.84 ± 3.68c	59.05 ± 4.40c
	% 3NB	89.34 ± 3.79ab	88.84 ± 13.02ab	80.40 ± 9.99b	60.71 ± 5.15bc	54.52 ± 1.41c	63.37 ± 9.32bc
	% 6NB	82.00 ± 7.22b	94.12 ± 8.77a	78.73 ± 6.71a	69.39 ± 1.84ab	66.33 ± 6.86ab	68.39 ± 8.32bc
	0	84.81 ± 2.99ab	73.94 ± 5.70b	70.71 ± 3.85ab	53.33 ± 2.66c	59.49 ± 3.41bc	65.99 ± 8.82bc
	% 3EB	88.03 ± 6.84ab	76.82 ± 6.66b	82.57 ± 14.80a	62.19 ± 5.49bc	72.11 ± 10.27a	86.02 ± 3.70a
	%6EB	93.37 ± 6.22a	82.67 ± 5.21ab	81.46 ± 8.87a	74.84 ± 8.84a	67.54 ± 3.90ab	76.66 ± 5.73ab
K %	0	4.05 ± 0.36ab	4.53 ± 0.29*ab	3.74 ± 0.77	3.41 ± 0.43a	3.46 ± 0.25ab	3.64 ± 0.04b
	% 3NB	4.50 ± 0.01*a	4.53 ± 0.31ab	4.03 ± 0.18	2.47 ± 0.76b	3.47 ± 0.59ab	3.45 ± 0.22*bc
	% 6NB	4.39 ± 0.27a	4.40 ± 0.22ab	4.48 ± 0.22	3.25 ± 0.26ab	3.03 ± 0.05bc	3.23 ± 0.16dc
	0	3.82 ± 0.33b	3.89 ± 0.26b	4.44 ± 0.41	3.22 ± 0.07ab	3.77 ± 0.62a	4.08 ± 0.11a
	% 3EB	4.07 ± 0.26ab	4.05 ± 0.47ab	4.28 ± 0.12	2.69 ± 0.29ab	2.79 ± 0.10bc	3.00 ± 0.12d
	% 6EB	4.07 ± 0.08ab	4.67 ± 0.42a	4.01 ± 0.32	2.71 ± 0.17ab	2.69 ± 0.08c	2.72 ± 0.19e
Mg %	0	0.12 ± 0.01c	0.14 ± 0.02b	0.15 ± 0.6	0.13 ± 0.02	0.14 ± 0.01a	0.14 ± 0.01a
	% 3NB	0.13 ± 0.01bc	0.15 ± 0.03b	0.14 ± 0.02	0.17 ± 0.07	0.14 ± 0.02a	0.14 ± 0.00*b
	% 6NB	0.13 ± 0.00bc	0.13 ± 0.01b	0.13 ± 0.01	0.12 ± 0.02	0.13 ± 0.01abc	0.14 ± 0.02a
	0	0.13 ± 0.02bc	0.13 ± 0.00b	0.15 ± 0.01	0.14 ± 0.02	0.14 ± 0.01ab	0.13 ± 0.01ab
	% 3EB	0.15 ± 0.01*bc	0.14 ± 0.02b	0.15 ± 0.01	0.12 ± 0.01	0.12 ± 0.02bc	0.12 ± 0.01ab
	% 6EB	0.18 ± 0.02a	0.20 ± 0.08a	0.18 ± 0.02	0.11 ± 0.01	0.11 ± 0.01c	0.11 ± 0.01b
Mn mg kg–1	0	203.59 ± 15.70a	156.26 ± 15.21a	119.38 ± 16.52a	173.98 ± 10.01b	167.08 ± 3.36b	147.71 ± 8.68a
	% 3NB	171.30 ± 9.28b	110.55 ± 7.48b	73.27 ± 2.66b	139.60 ± 12.38c	113.91 ± 7.30c	132.13 ± 35.27a
	% 6NB	130.05 ± 6.87c	101.41 ± 8.45b	57.68 ± 6.87c	135.63 ± 12.85c	101.04 ± 6.11d	95.11 ± 9.00b
	0	128.56 ± 17.80c	97.28 ± 13.11c	109.86 ± 2.83a	201.79 ± 11.07a	181.80 ± 4.25a	146.45 ± 10.42a
	% 3EB	72.45 ± 5.21d	64.18 ± 0.85d	55.05 ± 1.29c	100.86 ± 2.37d	92.64 ± 11.69d	85.43 ± 8.80b
	% 6EB	34.93 ± 1.70e	37.20 ± 0.53e	27.96 ± 4.10d	42.82 ± 4.69e	41.90 ± 2.74e	46.34 ± 3.39c
Na mg kg–1	0	63.21 ± 50.64c	64.83 ± 40.80c	420.76 ± 540.54 cd	1376.00 ± 0.01e	930.44 ± 37.06e	4044.11 ± 4479.90de
	% 3NB	384.53 ± 142.22c	348.04 ± 169.13c	632.69 ± 501.27 cd	8171.25 ± 306.72d	7432.99 ± 960.79d	7289.60 ± 570.48d
	% 6NB	960.72 ± 121.95c	615.49 ± 496.42c	1352.52 ± 226.65c	11763.62 ± 328.25c	14290.29 ± 929.42c	14290.06 ± 675.65c
	0	231.07 ± 48.00c	175.94 ± 60.04c	137.86 ± 84.22d	915.02 ± 40.43e	1029.21 ± 177.49e	1212.01 ± 402.23e
	% 3EB	3098.00 ± 699.78b	3756.40 ± 996.16b	3146.39 ± 1030.14b	22442.45 ± 1645.94b	24299.47 ± 1588.61b	23175.75 ± 2263.38b
	% 6EB	5853.41 ± 923.69a	5503.66 ± 1500.52a	5790.87 ± 435.13a	33785.89 ± 2417.15a	32475.42 ± 2309.53a	34704.23 ± 2565.03a
P %	0	0.35 ± 0.02ab	0.33 ± 0.04abc	0.23 ± 0.02c	0.28 ± 0.04	0.26 ± 0.04	0.23 ± 0.00
	% 3NB	0.37 ± 0.01*ab	0.34 ± 0.01ab	0.26 ± 0.01bc	0.24 ± 0.05	0.29 ± 0.08	0.30 ± 0.05
	% 6NB	0.39 ± 0.03a	0.37 ± 0.03a	0.30 ± 0.01ab	0.34 ± 0.03	0.31 ± 0.02	0.29 ± 0.04
	0	0.27 ± 0.05c	0.30 ± 0.03bbc	0.28 ± 0.05bc	0.28 ± 0.07	0.29 ± 0.04	0.26 ± 0.01
	% 3EB	0.32 ± 03bc	0.28 ± 0.04bd	0.30 ± 0.01ab	0.31 ± 0.01	0.31 ± 0.05	0.36 ± 0.02
	% 6EB	0.34 ± 0.02ab	0.37 ± 0.01a	0.35 ± 0.06a	0.35 ± 0.03	0.31 ± 0.02	0.37 ± 0.02
Zn mg kg–1	0	163.88 ± 14.56a	220.28 ± 25.51a	118.54 ± 41.13ab	95.37 ± 11.81ab	129.76 ± 9.29ab	148.28 ± 16.49a
	% 3NB	153.42 ± 1.04a	179.44 ± 13.71b	90.47 ± 13.94bc	78.83 ± 7.83bc	107.70 ± 22.52bc	137.68 ± 32.80a
	% 6NB	135.05 ± 11.76b	149.43 ± 8.41c	87.20 ± 9.41bc	91.82 ± 13.87ab	103.55 ± 5.71ab	131.50 ± 19.04a
	0	93.08 ± 14.18c	85.98 ± 5.80d	132.89 ± 5.06a	110.15 ± 10.67a	141.45 ± 18.03a	145.36 ± 12.13a
	% 3EB	65.23 ± 3.98d	75.95 ± 6.99d	91.11 ± 12.92bc	71.45 ± 6.72c	117.14 ± 16.73c	129.60 ± 9.45a
	% 6EB	57.28 ± 3.16d	69.85 ± 1.60d	67.91 ± 2.88c	77.14 ± 9.28bc	77.68 ± 2.86bc	92.81 ± 11.87b

a Data followed by different letters are significantly different according to Duncan’s Multiple Range Test (*p* < 0.05). Values without letters indicate no significant difference (*p* > 0.05).

### Durum Wheat

Table 3 shows the dry matter yield of durum wheat under different Cd and bentonite treatments. A statistically significant difference was found between the different Cd doses and the bentonite types (NB and EB) mixed into the soil (*p* < 0.05). The highest dry matter yield was obtained in the 6% EB treatment without Cd addition (710.32 ± 69.03 mg plant^–1^). However, no statistically significant difference was found between the bentonite applications and the control group at the other ratios. At Cd doses of 5 and 10 mg kg^–1^, the dry matter yield of the plants showed statistically significant variations (*p* < 0.05), with the highest values recorded in the 6% EB treatment (453.23–529.22 mg plant-^1^).

**Table 3:** Changes in dry Matter Weight of Both Wheat Varieties in Response to Cd Doses^a^

		dry weight (mg plant^–1^)		
cultivars	treatments	Cd 0	Cd 5 ppm	Cd 10 ppm
T. aestivum L.	% 0B	520.00 ± 16.17c	529.21 ± 62.78b	472.71 ± 14.36 cd
	% 3NB	696.67 ± 61.01b	735.42 ± 79.14a	576.79 ± 41.39abc
	% 6NB	749.75 ± 42.26b	760.42 ± 68.29a	634.04 ± 67.40ab
	% 0B	485.08 ± 55.51c	526.10 ± 98.38b	429.96 ± 102.79d
	% 3EB	700.46 ± 46.53b	669.14 ± 93.39a	515.67 ± 79.22bcd
	% 6EB	845.33 ± 31.75a	699.71 ± 38.35a	672.75 ± 55.99a
T. durum L.	% 0B	230.44 ± 49.34b	195.04 ± 18.00d	206.07 ± 29.41d
	% 3NB	210.13 ± 91.89b	247.54 ± 16.12 cd	306.29 ± 18.53c
	% 6NB	345.49 ± 91.22b	271.00 ± 27.26 cd	287.87 ± 18.07 cd
	% 0B	188.72 ± 61.45b	329.59 ± 69.84bc	280.52 ± 70.06 cd
	% 3EB	238.18 ± 76.85b	362.00 ± 13.44b	394.42 ± 71.95b
	% 6EB	710.32 ± 69.03a	453.23 ± 79.03a	529.22 ± 5.38a

a Data followed by different letters are significantly different according to Duncan’s Multiple Range Test (*p* < 0.05). Values without letters indicate no significant difference (*p* > 0.05).

**Figure 3:**
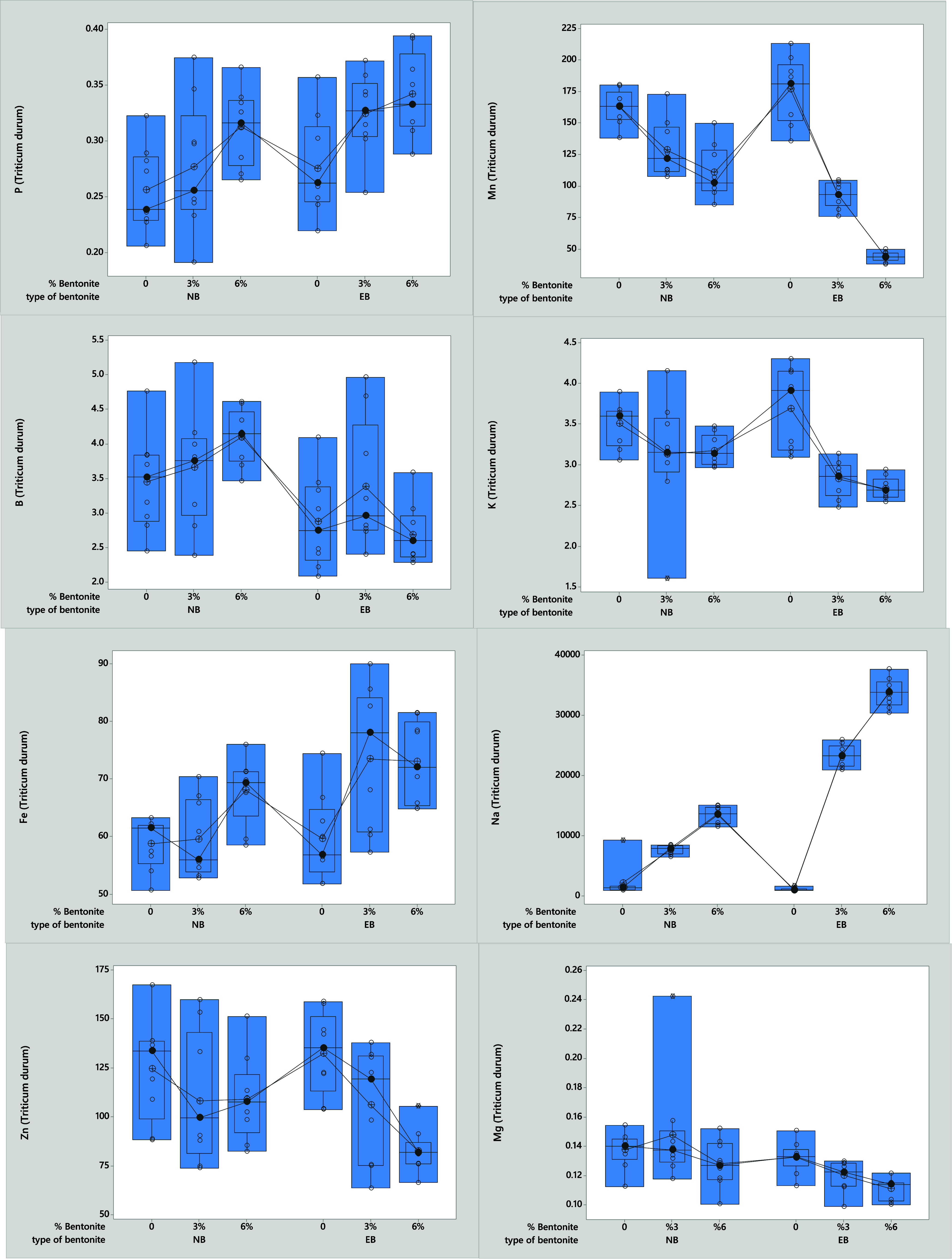
Effect of bentonite type and ratio on Cd accumulation in the shoot of *Triticum durum* L..

The effect of Cd and bentonite mixtures on the nutrient composition of durum wheat was statistically significant (*p* < 0.05). However, no significant difference was found between the bentonite-treated groups without Cd and the control group (Table 2, Figure 3). The highest B concentration was observed in the 6% NB mixture (4.14–4.19 mg kg^–1^). As for Ca concentration, the highest values were found in the statistically significant control applications (*p* < 0.05) (1.12–1.24–1.15%). While a significant difference was observed between the dose of 5 mg Cd kg^–1^ and the Cu concentration of the plants in the different bentonite mixtures, no significant difference was found at the dose of 10 mg Cd kg^–1^ (*p* < 0.05). The highest Cu concentration in the plants was found in the 6% EB treatment at 0 mg Cd kg^–1^ (10.33 mg kg^–1^), while at doses of 5 and 10 mg Cd kg^–1^ the highest Cu concentrations were found in the control group (11.08–11.35 mg kg^–1^).

The highest concentration of Fe was recorded at 74.84 mg kg^–1^ in the 6% EB treatment without the addition of Cd. In the presence of 5 and 10 mg kg^–1^ of Cd, the highest Fe concentrations ranged from 72.11 to 86.02 mg kg^–1^ in the 3% EB treatment. While the control group exhibited the highest K concentration at 3.41%, K concentrations were higher in the 6% EB treatment, measuring between 3.77 and 4.08% at doses of 5 and 10 mg Cd kg^–1^. Additionally, the 3% NB mixture had the highest Mg concentration, recorded at 0.17 mg kg^–1^, when no Cd was added. The highest Mn and Zn concentration was obtained in the control groups (201.79–181.80–146.45 mg Mn kg^–1^) (110.15–0.141.45–148.28 mg Zn kg^–1^). The highest Na concentration was determined in both Cd and non-Cd treated groups in the 6%EB mixture (33785.89–32475.42–34704.23 mg Na kg^–1^). The highest Na concentration was determined in Cd and non-Cd groups treated with 6% EB. (33785.89–32475.42–34704.23 mg Na kg^–1^). The highest concentrations of Mn and Zn were found in the control groups, with values of 201.79, 181.80, and 146.45 mg Mn kg^–1^ and 110.15, 141.45, and 148.28 mg Zn kg^–1^. The greatest concentration of Na was observed in both the Cd and non-Cd treated groups using a 6% EB mixture, yielding 33785.89- 32475.42- 34704.23 mg kg^–1^. Additionally, Figure 2 illustrates statistically significant differences in Cd concentration between plants and the bentonite mixture ratio (*p* < 0.05). While the highest Cd concentrations were recorded in the controls, with values of 3.48, 27.10, and 42.69 mg Cd kg^–1^, the lowest concentrations were found in the plants treated with 6% EB, showing values of 1.59, 13.05, and 22.08 mg Cd kg^–1^. Figure 2 shows statistically significant differences between Cd concentration in plants and bentonite mixture ratio (*p* < 0.05). Although the highest values for Cd concentration were obtained in the controls (3.48–27.10–42.69 mg Cd kg^–1^), the lowest Cd concentration was obtained in the plants with 6% EB (1.59–13.05–22.08 mg Cd kg^–1^).

## Discussion

Cd enters the soil through rock weathering and anthropogenic activities such as industrial emissions and phosphorus fertilizer applications. These heavy metals not only pollute the soil but also negatively impact crop productivity, quality, and food security. Moreover, heavy metals accumulated in soil contribute significantly to the contamination of agricultural products and pose potential health risks.^81,82^ Thus, Cd, which has a toxic effect on living organisms, is recognized as one of the most hazardous heavy metal pollutants in ecosystems.^83^ The remediation of Cd-contaminated agricultural soils aims to restore their agricultural value and ensure food security. One promising approach is the use of bentonite amendments, which have been shown to reduce Cd bioavailability by enhancing soil pH and transforming exchangeable Cd into more stable fractions, thereby limiting Cd uptake in plants.^84^ In addition, bentonite amendments have been reported to increase biomass production in crops by reducing oxidative stress and improving nutrient uptake efficiency.^85^ The aim of remediation of heavy metal contaminated agricultural soils is to restore their agricultural value and ensure food security. For this reason, the effects of bentonite on Cd-contaminated soils on plant growth, nutrients, and Cd concentration in wheat were investigated.

The findings of this study revealed that bread wheat grown in the 6% EB mixture had the highest dry matter accumulation in both Cd-treated and Cd-free conditions. Compared to the control, dry matter yield increased by 74.27% in Cd-free groups and by 56.47% at a Cd dose of 10 mg kg^–1^. However, at a dose of 5 mg Cd kg^–1^, the highest dry matter content was observed in the 6% NB mixture, which resulted in a 43.69% increase compared to the control. In durum wheat, the highest dry matter weight was recorded in 6% EB mixtures under Cd-free conditions, with an increase of 277.66% compared to the control groups. At 5 mg Cd kg^–1^, the increase was 37.51%, and at 10 mg Cd kg^–1^, it was 88.66%. This higher biomass accumulation in durum wheat (*T. durum*) under Cd stress can be attributed to its stronger metal sequestration and detoxification mechanisms, such as vacuolar compartmentalization, metal chelation, and an enhanced antioxidant defense system.^86^ Several studies have indicated that durum wheat possesses a higher Cd tolerance compared to other wheat species, primarily due to the activation of heavy metal transporters and metal-chelating proteins that help restrict Cd translocation to shoots and grains.^87^ These findings support the hypothesis that durum wheat exhibits adaptive tolerance to Cd stress, making it a viable crop for cultivation in Cd-contaminated soils. The greater increase in biomass observed in durum wheat (*T. durum*) under high levels of Cd can be explained by the tolerance and adaptation mechanisms that this species has developed in response to Cd stress. Although cadmium is a toxic heavy metal for plants, certain wheat species have developed a tolerance to Cd. Durum wheat in particular may have a higher tolerance to Cd stress. These results are consistent with the existing literature that emphasizes the role of bentonite in improving plant growth under heavy metal stress. Aydemir et al. showed that the application of lime and other additives can significantly affect the uptake of Cd in durum wheat cultivars, suggesting that soil additives can mitigate the negative effects of Cd on plant biomass.^88^ Similarly, Boorboori and Zhang reported that the application of silicon can increase the dry weight of wheat under Cd stress, suggesting that certain treatments can promote biomass accumulation despite the presence of toxic metals.^89^ The significant increase in biomass observed in durum wheat under high Cd stress can be attributed to its inherent tolerance mechanisms. Research has shown that certain wheat species, especially durum wheat, exhibit adaptive responses to Cd stress, which may include enhanced root growth and nutrient uptake. However, no specific evidence was found in the citations provided to support these claims. In addition, the study by Taha et al. highlighted that nitrogen fertilization can affect Cd availability and plant growth, suggesting that nutrient management plays a crucial role in mitigating the effects of Cd on wheat.^90^

The ability of durum wheat to maintain higher dry matter accumulation under Cd stress may also be related to its genetic and physiological characteristics that confer greater resilience to heavy metal toxicity.^91^ The results of this study emphasize the importance of bentonite and nutrient management in increasing dry matter accumulation in wheat under Cd stress. The significant increase in biomass observed in both bread wheat and durum wheat, especially in the 6% EB mixture, emphasizes the potential for the use of soil amendments to improve crop resilience in contaminated environments. This tolerance may also be achieved through increased antioxidant activity and increased production of enzymes that detoxify Cd. In addition, the genetic structure of durum wheat may have a higher resistance to Cd stress. This is also confirmed in the literature. For example, in their study on Cd accumulation and tolerance in different wheat varieties, Halim et al.^92^ found that certain varieties have a greater tolerance to Cd stress. It is known that heavy metals such as cadmium can trigger oxidative stress in cells.^93−95^ The higher biomass production of durum wheat under Cd stress could also be related to the stronger antioxidant defense mechanisms of this species. In addition, bentonite, a clay mineral with the ability to bind heavy metals in the soil, prevents cadmium from reaching the plant roots. In some cases, however, Cd can bind to the surface of bentonite and be taken up by the plant in a less toxic form. This could also explain the increased biomass of durum wheat in the presence of Cd. There were observable differences between the effects of natural and sodium-enriched bentonite. EB was more effective in reducing Cd uptake compared to NB. In both wheat varieties, the 6% EB mixture reduced Cd bioavailability and led to an increase in biomass. In particular, durum wheat exhibited a higher increase in biomass with the 6% EB application, indicating that EB acted as a more effective immobilization agent than NB.

Cadmium has also shown antagonistic interactions with various essential nutrients required for optimal plant growth.^96^ While the highest B concentration of 3% EB was obtained in the Cd-free groups, the highest B concentration of 6% NB mixtures was obtained at 5 and 10 mg Cd kg^–1^ doses (5.33–5.21) in bread wheat. These increases were observed at 0, 5, and 10 mg Cd kg^–1^ doses (15.5%, 39.53%, 16.56%) compared to the control groups. Similarly, increases were obtained with durum wheat (22.12%, 23.58%, 36.04%). In a hydroponic study, B, Si and their mixtures were reported to have a mitigating effect on Cd accumulation and toxicity in cultivated rice plants, reducing Cd-induced oxidative stress and Cd toxicity.^66^ It also prevents Cd from leaving the cell and chelates Cd to the cell wall.^97,98^ In addition, it contributes to the synthesis and cross-linking of B cell wall components.^99,100^ Therefore, the increase in B concentration in plants due to elevated doses of Cd may suggest that it may play a role in regulating Cd accumulation and toxicity in plant cells. It was observed that Ca concentration of both plant species increased with increasing Cd dose in control applications without bentonite. These increases were 14.67% and 25% of the 5 and 10 ppm of Ca concentrations of Cd administered to the control groups of bread wheat. Increases of 10.71% and 2.68% were also observed in durum wheat. The competing uptake of Ca and Cd is a crucial factor influencing the response of plants to Cd stress. Studies have shown that Cd can interfere with Ca uptake due to their similar ionic properties, which can lead to a decrease in Ca content in the plant when exposed to high Cd concentrations.^101^ This phenomenon is particularly important in the context of Cd toxicity, where the presence of sufficient Ca can mitigate some of the deleterious effects of Cd on plant health.^102^ For example, the addition of Ca has been reported to mitigate Cd toxicity by increasing the expression of calcium-transporting ATPases, which can help regulate the internal concentrations of these ions.^103^ In addition, the role of exogenous Ca in improving the tolerance of plants to Cd stress has been documented. Research suggests that Ca can increase photosynthesis and biomass accumulation, potentially diluting Cd concentrations in plant tissue.^104^ This is supported by evidence showing that increased Ca availability can lead to decreased Cd accumulation in various plant species, including wheat.^105^ The physiological mechanisms behind this include the regulation of metal uptake and translocation, which are critical for maintaining plant health under heavy metal stress.^106^ In the context of the specific increase in Ca concentration observed in bread wheat and durum wheat, it is important to consider the role of chelators and organic acids such as citric acid, which can improve the bioavailability of Ca and other nutrients in the soil-plant system.^107^ The use of chelators can affect the absorption of Ca and Cd, indicating that their presence in the soil may significantly influence the observed increase in Ca concentration following Cd exposure.^108^ Research has indicated that the application of Cd reduces Ca and Mg concentrations in the shoots of lettuce.^109^ In contrast, it increases Ca and Mg levels in both the roots and leaves of strawberries.^110^ Additionally, a study on rice found that Cd either negatively affected or had no significant correlation with Ca concentration.^111^ The negative relationship between Ca and Cd concentration could be due to competition between Cd^2+^ and Ca^2+^, as Cd^2+^ uses Ca channels to enter plant cells^112^ and may help inhibit Cd toxicity through photosynthesis by replacing Ca in proteins.

The uptake and translocation of minerals are essential for plant growth, and Cd can disrupt these processes, leading to various disorders.^113^ In both types of plants studied, there was instability in the concentrations of Fe, Cu, K, Mg, P, and Zn as a result of different doses of Cd supplied to the bentonite mixture groups. Cd is a nonessential metal that is absorbed by membrane transporters, which can interfere with the translocation and physiological functions of various nutrients.^114−118^ In a study with lettuce, Cd exposure was found to disturb the balance of N, P, K, Ca, Mg, and S, reducing their accumulation in shoots. In addition, a reduction in nutrient content was reported.^119^ This variation and inconsistency in nutrient concentrations in Cd-exposed plants has been linked to in situ Cd content and Cd detoxification mechanisms, among other factors.^120,121^ In both types of wheat, the Mn concentration in control crops was higher than in those treated with Cd and bentonite. The Mn concentration decreased by 5 and 10 ppm with increasing doses of Cd for both bread wheat (23% and 41% decreases) and durum wheat (11% and 27% decreases). A negative correlation was observed between Cd doses and Mn concentration in both wheat cultivars. However, while this correlation was statistically significant for bread wheat (*p* < 0.05), it was not significant for durum wheat. Furthermore, varying the percentage of bentonite in the mixture resulted in a highly significant negative correlation with Mn concentration in bread wheat (*p* < 0.001). Durum wheat also showed a statistically significant negative correlation for Mn concentration (*p* < 0.05). The detrimental effect of Cd on Mn uptake can be attributed to the competitive interactions involved in the uptake of metal ions by plants. Studies have shown that elevated Cd concentrations can inhibit the uptake of essential micronutrients, including Mn, due to their similar ionic properties.^122^ This is particularly evident in wheat varieties, where the presence of Cd in the soil can lead to reduced Mn availability and uptake, ultimately affecting plant health and yield.^123^ In addition, the presence of bentonite, a clay mineral, has been reported to impair the bioavailability of nutrients in soil, potentially exacerbating the negative effects of Cd on Mn concentrations.^124^

Statistical analysis has revealed a significant negative correlation between Cd dose and Mn concentration in bread wheat. This indicates a strong relationship where increased Cd levels lead to reduced Mn content. In contrast, the lack of statistical significance observed in durum wheat suggests that this variety may respond differently to Cd exposure, potentially due to genetic or environmental factors.^125^ The reactions of these wheat varieties underscore the importance of understanding crop-specific interactions with heavy metals, which is essential for developing agricultural practices to mitigate soil contamination.^126^ Additionally, the incorporation of bentonite has demonstrated a significant negative impact on Mn concentration in bread wheat (*p* < 0.001). A significant negative correlation was also noted in durum wheat (*p* < 0.05), suggesting that adding bentonite to the soil could complicate nutrient uptake dynamics in the presence of heavy metals. This emphasizes the need for careful management of soil amendments in agricultural systems.^127^ Furthermore, the availability of Mn to plants decreases when Cd is present in the soil.^121^ A regression analysis indicated a significant negative correlation between Cd and Mn, highlighting the antagonistic effect of Cd on Mn uptake and translocation.^128^ In summary, the observed decreases in Mn concentrations in both bread wheat and durum wheat in response to Cd exposure and bentonite application underscore the complex interactions between heavy metals and essential micronutrients in plant physiology. Understanding these relationships is critical for developing strategies to enhance nutrient uptake and mitigate the detrimental effects of soil contamination on crop production.

There was no statistically significant difference between Na concentration and Cd dose in wheat cultivars, bread wheat and durum wheat. However, a statistically significant positive relationship was found between bentonite applications and plant Na concentration (*p* < 0.001). The highest Na concentration in both wheat cultivars was obtained at 6% EB. After 0, 5, and 10 mg Cd kg^–1^, Na concentration in both wheat cultivars decreased in the control groups, while plant Na concentration increased by 6% in the EB mixtures. These increases were observed in bread wheat (2434%, 3045%, 4126%) and durum wheat (3592%, 3056%, 2763%). Research indicates that various environmental factors, including soil additives like bentonite, can affect the accumulation of sodium in plants. Bentonite, a clay mineral, can increase the retention of nutrients and improve soil structure, which can facilitate increased Na uptake in plants.^129^ The significant increase in Na concentration observed in both bread wheat and durum wheat at higher bentonite levels emphasizes the role of soil amendments in nutrient dynamics and plant health.^130^ In relation to Cd loading, the data indicate that Na concentrations decreased in both wheat cultivars in the control groups treated with 0, 5, and 10 mg Cd kg^–1^. This finding aligns with previous studies showing that heavy metals like cadmium can hinder the uptake of essential nutrients, including sodium. This impairment occurs due to competitive interactions at the root level.^131^ The negative effects of Cd on Na concentration can be attributed to the physiological stress caused by the metal, which can lead to altered ion transport mechanisms in plants.^132^ The observed increases in Na concentration in response to bentonite mixtures (2434%, 3045%, 4126% for bread wheat and 3592%, 3056%, 2763% for durum wheat) emphasize the potential of bentonite to mitigate the negative effects of Cd by improving nutrient availability. This is consistent with evidence suggesting that soil amendments can improve plant resilience to heavy metal stress by promoting better nutrient uptake and overall plant health.^133^ The significant differences in Na concentration due to bentonite applications suggest that such amendments could be a viable strategy to improve the nutrient profile of wheat crops on contaminated soils.^134^ Na concentration in both bread wheat and durum wheat did not show statistically significant differences at different Cd doses, but the positive correlation with bentonite applications suggests the importance of soil amendments to improve nutrient uptake. The increase in Na concentration observed at higher bentonite levels suggests that these amendments may play a critical role in improving plant health and mitigating the effects of heavy metal stress. In one study, NaCl application on leaves was found to increase the accumulation of Cd in *T. durum* L. compared to the control groups.^135^ Cd and Na may share some common channels to enter a cell and compete for Cd binding sites that are not taken up. Therefore, it can be hypothesized that this may lead to a decrease in Cd uptake.^136^

It was also found that the application of clay minerals, especially Ca-bentonite and Na-bentonite, reduced the availability of Cd, Cu, Zn, and Ni in soils contaminated by sewage sludge.^137^ Many studies have shown that bentonite can significantly inhibit the exchangeability of Cd in soil.^138^ In this study, the concentration of Cd in the plants was negatively correlated with the type of bentonite used and the mixing ratio, although no statistically significant difference was found. At cadmium doses of 5 and 10 ppm, the lowest Cd concentrations were observed in both wheat varieties with 6% EB mixtures. The reduction in Cd levels for the two types of wheat was 55% and 66% for bread wheat, and 55% and 48% for durum wheat compared to the control groups (Figure 2). These results suggest that the 6% EB mixture effectively reduces Cd uptake in the plants. Similar studies reported that bentonite reduced Cd uptake in rice, corn, and vegetables.^66,67^ Natural bentonite is a strong absorbent that has been used to adsorb heavy metal ions and organic matter in several recent studies.^139^ Cd was mostly converted to a residual fraction and this decreased Cd availability was mainly caused by metal absorption to the tectosilicate.^73,84^ It has been observed that the use of bentonites leads to the immobilization of Cd in soil and a decrease in its bioavailability. Therefore, the mechanisms of immobilization of Cd have also been attributed to the inhibitory effect of uptake by crops.^140^ Research has shown that bentonite can effectively reduce the bioavailability of heavy metals, including Cd, in the soil-plant system. For example, Klik et al. demonstrated that applying bentonite significantly decreased the uptake of cadmium and zinc in the above-ground parts of the tested plants. This suggests that bentonite enhances the immobilization of heavy metals and reduces their bioavailability.^141^ This finding aligns with Desalegn et al., who noted that the adsorption of Cd (II) onto bentonite composites is influenced by pH and the presence of functional groups, which can improve the binding of cadmium and decrease its uptake by plants.^142^ Additionally, the study by Jakfar and Azwar highlighted that using natural bentonite can optimize the adsorption of cadmium ions, underscoring the effectiveness of bentonite in reducing Cd concentrations in crops.^143^ The results from the current study support these findings, as a 6% bentonite mixture led to significant reductions in cadmium uptake in both types of wheat. This indicates that bentonite not only immobilizes cadmium in the soil but also plays a critical role in enhancing plant health by minimizing the toxic effects of heavy metals.

Furthermore, research conducted by Wyszkowski supports the idea that bentonite amendments can significantly reduce the mobility of trace elements, including cadmium, in contaminated soils. Their study reported a decrease in cadmium levels in the soil, which consequently resulted in lower concentrations in plant tissues.^144^ This reinforces the conclusion that incorporating bentonite into agricultural practices can effectively manage heavy metal contamination in crops.

In summary, the use of bentonite, especially at a mixing ratio of 6, effectively reduces the uptake of Cd in both bread wheat and durum wheat. The observed negative correlation between Cd concentration and the use of bentonite underlines its potential as a soil additive to improve the safety and quality of crops grown in contaminated environments. This study was conducted under controlled greenhouse conditions. Although the results provide valuable insights into the physiological responses of durum wheat to Cd stress, caution should be exercised when applying it in the field. Soil composition, environmental factors, and long-term effects of bentonite application may differ significantly from the controlled environment used in this study. Therefore, further field trials are required to validate the effectiveness of bentonite in reducing Cd uptake and to assess its wider implications for agricultural practice. Using bentonite as a soil additive to reduce Cd pollution is very promising for practical application in agriculture. By reducing Cd uptake in crops, bentonite could improve food safety and reduce the risk of heavy metal accumulation. However, the long-term effects of bentonite on soil health and structure need to be carefully assessed. Excessive use of clay minerals such as bentonite can lead to soil compaction, reduced water infiltration and changes in soil microbial communities, which could affect plant growth over time. In addition, the cost-effectiveness of large-scale use of bentonite should be considered. Although bentonite is relatively inexpensive, its long-term use and potential effects on soil structure and fertility require further research to ensure sustainable agricultural practices.

This study shows that durum wheat can tolerate higher levels of Cd and that bentonite plays an important role in reducing the bioavailability of Cd in contaminated soils. However, the long-term sustainability of soil amendments such as bentonite, particularly concerning their effects on soil structure and plant health, needs to be further investigated. Future research should focus on field trials to assess the wider applicability of these results and explore the possible balance between reducing Cd uptake and maintaining soil quality.

## Conclusion

This study demonstrated that the application of natural and sodium-enriched bentonite effectively reduced the bioavailability of Cd in both bread wheat (*T. aestivum* L.) and durum wheat (*T. durum* L.). This reduction in bioavailability led to decreased Cd uptake and improved plant growth under Cd-contaminated conditions. Specifically, the application of 6% sodium-enriched bentonite was the most effective, resulting in a 66% reduction in Cd concentrations in bread wheat and a 48% reduction in durum wheat compared to control groups.

Additionally, the use of bentonite significantly increased biomass production, particularly in durum wheat, which exhibited an 88.66% increase in dry matter weight under Cd stress at a concentration of 10 mg kg^–1^ with the application of 6% sodium-enriched bentonite. The results also revealed species-specific differences, with durum wheat showing greater biomass accumulation under Cd stress, likely due to its inherent tolerance mechanisms.

Furthermore, the findings indicated that sodium bentonite has high potential for reducing the presence and toxicity of heavy metals in contaminated soils. A negative correlation was observed between the Cd concentration in the plants and the amount of bentonite applied to the soil. This suggests that applying bentonite in agricultural areas contaminated with Cd could immobilize the metal in the soil, offering a viable strategy for growing crops in heavy metal-contaminated environments.

## Data Availability

Data is provided with in the manuscript file.
